# Combination of Blockchain and AI for Music Intellectual Property Protection

**DOI:** 10.1155/2022/4482217

**Published:** 2022-04-28

**Authors:** Na Li

**Affiliations:** Zhengzhou Preschool Education College, Zhengzhou, Henan 450000, China

## Abstract

In the last two years, due to the pandemic and restrictive measures, the dependence of music creators and artists on the Internet, where they could promote their work, organize live streaming concerts, and talk to the public, has increased and expanded even more and seeks higher revenue from digital music platforms. An important issue that arises from the above statement is protecting the authors' copyright regarding the uses and sharing in the digital services of their works with protected content. Although circulated in digital information, the protected content is not information but a product of ethical and commercial value. While it has an intangible owner and it owes its existence to the creative idea of its creator, it is not an idea. The imposition of legal and commercial conditions on its movement cannot be associated with any restrictions on the free movement of information, as it is not related to them. In general, the unauthorized exchange of digital music files via peer-to-peer violates copyright law. The exchange of files is unauthorized, as it does not have the relevant permission from the creators and beneficiaries and is therefore illegal. With this in mind, this paper proposes a highly effective way of protecting the copyright of music technology, which is based on the widespread use of artificial intelligence, blockchain, and cryptography technologies. Specifically, an advanced blockchain model based on Hyperledger Fabric is introduced, which, however, uses Quantum Homomorphic Encryption and Quantum Zero-Knowledge Arguments. Music files are implemented as Nonfungible Tokens (NFTs), which activate smart contracts. Finally, an advanced collaborative filtering algorithm provides recommendations for effectiveness in securing the copyrights of music industry creators. A specialized scenario was built to model the proposed system to verify the degree of protection on music intellectual property in developing a security simulation with an innovative consensus-based zero knowledge and the quantum fully homomorphic encryption technique. Experiment results show that this technique can aid in implementing a technologically aware system capable of providing a powerful answer to a current real-world problem.

## 1. Introduction

Developments in the digital realm are rapid and have a strong imprint on the livelihood of songwriters and musicians. Through digital music services where their music is played, artists are now looking for income, especially in the wake of the pandemic and the restrictive social distancing measures that have been imposed. It was an alternative artistic outlet for the music industry. It turned to the Internet and music and movie/series streaming services as the dominant entertainment solution in combination with the public. These platforms have made huge profits, while on the contrary, the income of musicians from live concerts and, in general, the income from digital uses is still limited. In addition, piracy, and the sharing of music archives in general, without respect for copyright, is a massive escape from profits. Under copyright and related rights law, copying, translating, performing, or presenting a piece of music to the public, or posting a piece of music on the Internet, is illegal unless there are specific exceptions to the law or permission from all beneficiary parties [1, 2]. In this context, various organizations have moved dynamically in all directions, with business agreements, beneficial collaborations, political means and institutional demands, communication campaigns, and actions to promote the interests of the creators and to highlight their constant need for respect for the copyrights of musicians by all without any exception [1, 3].

The unauthorized peer-to-peer exchange of digital music files violates copyright law. File exchange is unauthorized since it does not have the necessary consent from the creators and beneficiaries and hence is prohibited. The best solution for protecting the musical rights of creators and beneficiaries in the rapidly evolving landscape of the global digital world that affects the field of copyright imposes advanced and technologically up-to-date solutions [4]. New specialized tools are required using advanced algorithms, which will help to identify, in addition to the original declared uses, the musical works in the digital services and the Internet. This will contribute firstly to protecting their copyrights and secondly to the further increase of musicians' income from their works' online and multidisciplinary uses [5, 6]. Multiterritorial online licensing involves licensing music works on the Internet and, to giant users, the so-called digital or online music service providers covering more than one national and geographical territory with various legal approaches [7].

Wanting to overcome the severe weaknesses that characterize traditional mechanisms, this work proposes a highly effective way of protecting the copyright of music technology, which is based on the widespread use of artificial intelligence, blockchain, and cryptography technologies [8]. This template provides a complete cycle of services that start by lending the represented musical works to the online services, locating, and identifying them in the usage reports, collecting the corresponding rights, up to the detailed liquidation of rights that will be in the hands of the creators and beneficiaries of the temporary or total assignment of their copyright. The above stages of this integrated service are encoded in Nonfungible Tokens (NFTs) [9], activated by smart contracts in the Hyperledger fabric [10, 11]. They use advanced quantum encryption methods in the consent mechanisms and the blockchain protection itself. Specifically, an enhanced blockchain paradigm based on Hyperledger Fabric is presented, but with the addition of Quantum Homomorphic Encryption and Quantum Zero-Knowledge Arguments. NFTs are used to implement music files and to activate smart contracts. Finally, a specialized collaborative filtering algorithm makes intelligent recommendations for efficiency and transparency in the use of music content, the detection of violations of preagreed copyright protection rules of music works, the possibility of correcting errors by modifying smart contracts, and the distribution based on actual use [12]. This is a sophisticated system that is proposed for the first time in the literature.

The research is structured as follows.  Section 2 provides a complete overview of the topic's associated review. The proposed prototype is shown in Section 3.  Section 4 contains a concept of proof in a relevant scenario, and the final section draws conclusions and suggests future study directions.

## 2. Related Literature

With the development of the Internet, online content has been tremendously increased. In recent years, the research community has focused on the practical use of blockchain technology [13–15].

Li et al. [16] introduced a blockchain-based decentralized music copyright operation management system that disconnects the link between activities and property. They created a distributed licensing management system utilizing the blockchain's shared ledger system and smart contracts as the Ethereum platform's main structure [17]. The platform also brings together the concerns of artists, producers, owners, and customers, which benefits the audio sector's future growth and provides users with more meaningful content and a greater outcome. According to them, the platform is still in the development and upgrading stages.

Bakhytzhan et al. [8] looked at innovative ways to legal control audio media in particular and difficulties and answers. They emphasized a common flaw in the law: it does not follow up with technological advancements and possibilities to manage these relationships. They claimed that the deployment of blockchain technology confronts significant challenges and that many legal concerns must be overcome for blockchain to operate and grow as a property control instrument. The preservation of legal items in digital format is one of the possible applications of blockchain [4]. However, there are still specific issues in the digital music business relating to licensing and distribution, royalties paid by the patent owner, and illicit copying. They concluded that blockchain and smart contracts might help with author rights management in two ways: first, by automating the whole process of creating a licensing agreement, and second, as interim measures of the parties' duties, such as royalties' payment.

Ito and O'Dair [18] outlined the critical obstacles to managing artistic ownership in the digital age before looking at how advocates of the ledger and public blockchain technologies have proposed that these technologies help solve these problems. They also looked at the most critical technical terminology. They concluded that blockchain is a cutting-edge technology that has the potential to revolutionize intellectual property management. A tight emphasis on the technology itself, on the other hand, might lead to a lack of awareness of significant operational and implementation issues. The right design of incentives, at both the operational and implementation levels, will be critical to the efficient use of blockchain software for intellectual asset administration.

Gurkaynak et al. [19] concentrated on the potential benefits of blockchain for the development of intellectual property law and its implications for the registration, maintenance, and protection of creative intellectual assets. They extensively explained the idea of blockchain, examined the hurdles and impediments, detailed the legal position of blockchain and its possible use in intellectual asset law, and outlined the issues. They finished by making recommendations to help progress blockchain technology and raise the number of people aware of it, as well as its effective integration into different services and registration/transaction scenarios.

Amelin et al. [4] looked at employing blockchain technology to keep track of intellectual property items. One of blockchain's main drawbacks is the absence of full-fledged systems for settling creative ownership issues. This issue necessitates not just technological solutions but also institutional and legal ones. According to the authors, separating such registers into two categories has implications for the creation of blockchain systems for registering and maintaining intellectual property. The first is the permissionless blockchain platform's registers of intellectual property items. The information about entities and their users will not be authorized. The second kind is IP object registries based on license blockchain technology, in which government agencies with IP protection authority will be given superuser powers. They will have formal entries with validating rights value. They claim that the benefits of adopting blockchain-based registers outweigh the disadvantages of centralized registries and that they will be in high demand for securing intellectual property objects in general.

From the above literature, we conclude that even though the research community has conducted a significant study, the practical solutions provided for the effective implementation of blockchain are still limited [7, 8].

## 3. Proposed Prototype

Hyperledger is a connected P2P network system for developing decentralized ledgers based on blockchain technology. The proposed implementation is based on the architecture of Hyperledger Fabric (HyFa) [10, 20] to establish a system that will contribute to collaboration between various companies, organizations, and stakeholders without the use of public blockchain applications. The commonly accepted HyFa platform combines individual independent sections, which can be selected and combined to provide a solution to protect the music rights of authors and beneficiaries [14, 21, 22].

Consensus in the proposed framework is divided into the following:

1. In Endorsement, guided by a policy pursued by the participants to approve a transaction
2. In Ordering, which accepts the approved transactions and agrees on whether the proposal can be registered in the ledger
3. In Validation, which receives a block of orders and validates the correctness of the result, including the control of the validation policy and the double charge

**Figure 1:**
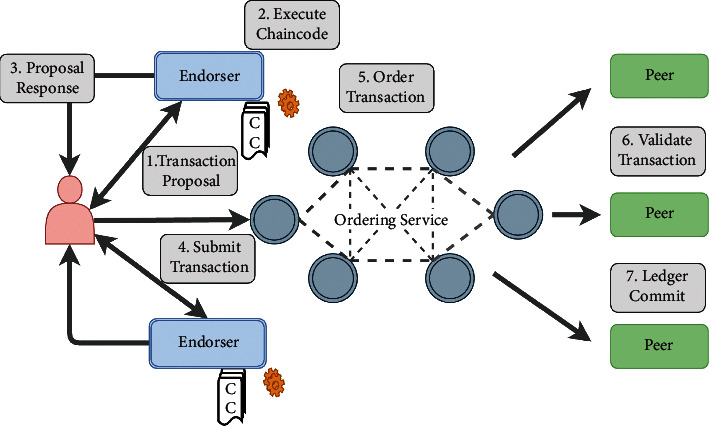
Prototype-high level transaction flow.

Each transaction or update of the ledger follows the flow of Figure 1 [20].

The client uses the interface programming environment to form a transaction proposal, which includes the channel name, the chained code name, and the input parameters of the code to be executed. The customer then sends a transaction proposal to all prospective escorts to satisfy the validation policy of that chain code [5, 23]. The peers simulate the transaction based on the parameters received by the customer, interacting with the chain code to record the updates and produce results in the form of a read and write set following the set signature and returning the results to the customer. The client collects the answers from all the peers and confirms that the results are consistent, e.g., all candidates who have subscribed to the same payload. The following is the merging of all the peers' signatures together with the registration readings and the creation of a transaction that is submitted to the ordering service [4, 7, 14].

The proposed ordering service innovates using a consensus algorithm based not on the standard Byzantine Fault Tolerance mechanism but on a zero-knowledge quantum protocol for the QMA class. To achieve zero knowledge, we use the nonblack-box extraction technique that allows the simulator to “imitate” the honest prover without knowing the witness. The protocol consists of a fixed number of rounds (>4) and achieves computational zero-knowledge. We try to attain statistical zero-knowledge while at the same time reducing the number of rounds to four. The proposed implementation requires a fully homomorphic quantum system, which allows homomorphic calculations in quantum circuits and messages [24, 25]. We also use compute-and-compare obfuscation. A compute-and-compare program CC [*f*, *s*, *z*], where *f* is a function and the *s*, *z* strings, has as output the value *z* for each input *x* where *f*(*x*) = *s* while rejecting any other entrance. A compute-and-compare obfuscator converts the CC program to the obfuscate CCg program, where it is computationally indistinguishable from a simulated “dummy” program that rejects all inputs. Finally, we use a Conditional Disclosure of Secrets (CDS) protocol. A CDS protocol consists of two rounds and has a sentence *z* and a message *m* from the sender as input. The recipient receives *m* only if the sentence is true; otherwise, the message remains hidden. At the same time, the witness *w* of the recipient's motion *z* remains hidden from the sender [26, 27].

Then, we use two rounds of Witness-Indistinguishable Arguments (W-IA), which is the basis of the following results. The protocol consists of a commitment *α*, a challenge *β*, and a response *γ*. The valuable property for us is the calculation of *β* and *γ* with an additional message (from the verifier to the prover) to achieve statistical zero-knowledge. The main idea of the protocol is to use a (leveled) fully homomorphic cryptosystem with maliciously circuit private security to reduce the rounds as the verifier sends an encrypted challenge *β* and the prover first calculates the binding *α* and then the answer *γ* is homomorphically encrypted. Knowing the private key of the homomorphic cryptosystem, the verifier can decrypt the ciphertext it receives and confirm they are the right ones (*a*, *b*, *c*). All the above can be constructed considering the quasi-polynomial difficulty of the LWE problem [28, 29].

Cryptography plays a significant role in the proposed model, providing tools that enable secure communication between two or more individuals and securing copyright protection. In particular, the proposed system bypasses the widely known cryptographic protocols, such as public-key encryption, using a quantum fully homomorphic rate-1 encryption scheme, which allows the calculation of functions with encrypted data input for its secure communication via public channels [30, 31].

A fully homomorphic encryption system allows one party to send its encrypted message *m* under a public key so that the other can then, having a *C* circuit, calculate and send [32]:

(1) $$\begin{array}{c} \text{Enc}\left(m\right)\overset{\text{Eval}\left(C,\cdot \right)}{⟶}\text{Enc}\left(C\left(m\right)\right), \end{array}$$

without learning any new information about the message *m*. Computations on encrypted data apply when a computer-weak client wants to upload his data to a more powerful server that can run complex circuits while maintaining his privacy. The proposed template goes a step further and uses a quantum fully homomorphic rate-1 encryption scheme while ensuring that the communication complexity introduced by the protocol does not negate the enhanced performance offered by the server.

Before delving into our proposal, it is essential to understand why existing protocols fail and show a more significant rate. The rate is generally defined as the fraction of the magnitude of the result of the calculations in nonencrypted information concerning the magnitude of the impact of the measures in encrypted information. In quantum protocols, a cryptocurrency that encrypts a *ℓ*-qubit quantum state |*ψ*〉 is in the following form [30, 32, 33]:

(2) $$\begin{array}{c} \text{QOTP}\left(\left(x_{1},z_{1},\ldots ,x_{\ell },z_{\ell }\right),|\psi \rangle \right),\text{QEnc}\left(\text{pk},\left(x_{1},z_{1},\ldots ,x_{\ell },z_{\ell }\right)\right), \end{array}$$

where QOTP (Quantum One-Time Pad) is applied separately to each qubit and the string otk = (*x*_1_, *z*_1_,…, *x*_*ℓ*_, *z*_*ℓ*_) is encrypted bit by bit. It is easy to notice that this cryptosystem has an inverse polynomial rhythm due to its classic homomorphic rate. An obvious solution would be to adopt a hybrid approach and sample the QOTP key using a pseudorandom generator. More specifically, we could improve the pace by calculating [34, 35]

(3) $$\begin{array}{c} QOTP\left(PRG\left(seed\right),\vee \psi \rangle \right),QEnc\left(pk,seed\right), \end{array}$$

for some uniformly sampled seed ←$ {0, 1}^*λ*^. Then, we can still calculate a function with encrypted input since there is the possibility of converting the ciphertext to its original form, homomorphically calculating the pseudorandom generator. Although this approach works for ciphertext that has just been encrypted, the same is not valid after a homomorphic application of functions; depending on the gateway to be applied to the quantum input, the QOTP otk key changes to a different otk′ string. Although there is a way to refresh the classic part of the ciphertext with each calculation, it does not fit with the hybrid approach we are considering. This is because the modified otk′ probably does not belong to the arrival set of the pseudorandom generator PRG; i.e., there may not be a seed′ string such that PRG (seed′) = otk′. Therefore, we observe that we cannot eliminate the classic QEnc (pk, otk) encryption. Even in the ideal case where the classical homomorphic shape has an optimal rate since at least two classical bits are required to encrypt a qubit, we will still have more complexity than desired and reach a dead end. So, the proposed quantum fully homomorphic rate-1 encryption scheme is ideal in terms of both security and performance [36–38].

The use of copyright will be based on Nonfungible Tokens (NFTs), which will be activated using smart contracts. Each NFT is a digital asset stored on a secure but transparent global blockchain. It is also nonexchangeable, which means unique. Thus, an NFT is a digital component associated with a distinct and unique component. NFTs contain security, transparency, and unchanged encryption storage, are indivisible, and can store significant amounts of data, including individual information. This is what makes a particular distinctive nonexchangeable and stored in a Smart Contract, which is code that executes automatically when a set of conditions is displayed [39, 40].

Combining a Smart Contract with other unique identifying metadata—such as the owner's identity and secure file links—along with the security provided by the blockchain provides virtually unquestionable proof of ownership and authenticity to potential buyers. Smart Contracts can prevent someone from transferring an NFT or accessing an underlying asset unless all the conditions set out in the contract are met, including the possible payment of royalties for the resale of the NFT. The NFTs in this standard are also coded to enforce Smart Contract copyright clauses. When reselling an NFT, automatically pay a fee to the seller, usually a fixed percentage of the resale price [41, 42].

In conclusion, NFTs and smart contracts act as certificates of authenticity to the underlying asset and as a valuable representation of the ownership of a tangible asset as a stock. The NFT holder has access to the underlying asset but may not have exclusive access to or control of the asset, let alone the asset owner or any intellectual property. The default rule is that the patent holder or copyright holder retains all intellectual property rights unless it is clear from the terms of the market (e.g., the Smart Contract in our case) that ownership of a copyright is being transferred [24, 42].

Finally, the template uses specialized collaborative recommendation mechanisms to make intelligent recommendations for efficiency and transparency in the use of music content, detect breaches of preagreed copyright rules, and correct errors by modifying smart contracts and based on actual usage. Specifically, a memory-based collaborative filtering methodology is used, which calculates the usefulness of each object for a user by directly processing all the evaluations contained in the system. For example, if *I*_*au*_ is the set of objects shared by users *a* and *u*, then their degree of similarity can be deduced from the Minkowski distance [19, 43, 44]:

(4) $$\begin{array}{c} w_{a,u}\equiv d_{a,u}={\left[\sum_{i\in I_{au}}{\left|r_{a,i}-r_{u,i}\right|}^{k}\right]}^{1/k}, \end{array}$$

where *k* is the class of the distance. For various values of *k*, the general formula of the equation takes specific forms. The cosine factor is also used:

(5) $$\begin{array}{c} w_{a,u}=\frac{\overset{⟶}{I_{a}\cdot \overset{⟶}{I_{u}}}}{{\left\|\overset{⟶}{I_{a}}\right\|}_{2}\cdot ||{||}_{u}}=\frac{\sum_{i\in I_{au}}r_{a,i}r_{u,i}}{\sqrt{\sum_{i\in I_{au}}r_{a,i}^{2}}\sqrt{\sum_{i\in I_{au}}r_{u,i}^{2}}}, \end{array}$$

and the Pearson correlation coefficient:

(6) $$\begin{array}{c} w_{a,u}=\frac{\sigma \left(I_{a},I_{w}\right)}{\sigma_{I_{a}}\times \sigma_{I_{w}}}=\frac{\sum_{i\in I_{au}}\left(r_{a,i}-r_{a}\right)\left(r_{u,i}-r_{u}\right)}{\sqrt{\sum_{i\in I_{au}}{\left(r_{a,i}-\bar{r_{a}}\right)}^{2}\sum_{i\in I_{au}}{\left(r_{u,i}-\bar{r_{u}}\right)}^{2}}}, \end{array}$$

where *σ*(*I*_*a*_, *I*_*w*_) is the covariance of the user's scores *a* and *u* and *σ*_*Ia*_, *σ*_*Ib*_, the corresponding standard deviations.

In the final stage, the recommendation type estimates a particular object's benefit for a given user. Its general form is shown in the following equation:

(7) $$\begin{array}{c} \hat{r_{a,i}}=\underset{u\in U_{a,i}}{\text{aggr}}r_{u,i}, \end{array}$$

where $\hat{r_{a,i}}$ is the value of the benefit of the object *i* for the user *a* or the prediction of the evaluation that the user *a* would make for the object *i*, if he had known in the past. *U*_*a*,*i*_ is a set of “similar” to *a* users (or objects) who have already evaluated object *i* and which has been created by the filtering process. The aggr notation describes how peer-to-peer ratings are processed. The relative formulas used are the simple average:

(8) $$\begin{array}{c} \hat{r_{a,i}}=\frac{1}{\left|U_{a,i}\right|}\underset{u\in U_{a,i}}{\sum }r_{u,i}, \end{array}$$

the weighted average:

(9) $$\begin{array}{c} \hat{r_{a,i}}=\frac{\sum_{u\in U_{a,i}}w_{a,u}r_{u,i}}{\sum_{u\in U_{a,i}}w_{a,u}}, \end{array}$$

and the Resnick type:

(10) $$\begin{array}{c} \hat{r_{a,i}}=\bar{r_{a}}+\frac{\sum_{u\in U_{a,i}}w_{a,u}\left(r_{u,i}-\bar{r_{u}}\right)}{\sum_{u\in U_{a,i}}\left|w_{a,u}\right|}. \end{array}$$

The main advantages of the process are related to the ease of their algorithmic implementation and the possibility of immediate update of the forecasts as soon as new (or existing) ratings are added to the system. Also, the generated forecasts are constantly improving with the increase of the available ratings in the system.

## 4. Proof of Concept

For the modeling of the proposed system, a specialized scenario was implemented to verify the degree of protection on music intellectual property in implementing a security simulation with an innovative consensus-based zero knowledge and the quantum fully homomorphic encryption scheme [12, 27, 45].

Specifically, the validity of the scenario is a direct consequence of the security of the homomorphic cryptosystem, to prove which of the following changes are required:

1. The prover calculates a random commitment used in the homomorphic calculation. Thus, the verifier can (in proof of correctness) confirm the validity of the protocol without knowing the private key of the homomorphic cryptosystem. While maintaining WI statistics, a particular commitment scheme is used, the Sometimes Binding Statistically Hiding (SBSH) commitment. There is a (negligibly small) chance of having a perfect commitment in such a commitment scheme. In this case, the verifier can export the blocked message. The leveraging technique proves the correctness.
2. All the above procedure is repeated twice, and the prover proves that in at least one of the two cases, the calculations were done correctly, using a WI statistic (for the NP class). This is enough to prove the nondiscrimination of the witness of the total protocol since in the proof we can “exchange” the witness in each step separately.

Since this protocol is the basis for the following, those, in turn, will be based on the quasi-polynomial difficulty of the problem.

In the simulation technique, for simplicity, we first consider verifiers that do not interrupt communication and are explainable; i.e., honest verifier algorithms support the messages. The essence of the protocol is the exportable commitment scheme which works as follows [19, 46]:

1 The sender samples two random strings *s*, td, and
  - A public and a private key (pk, sk) of a homomorphic QFHE cryptosystem and the string encryption td, *c*_td_ = QFHE.Enc(pk,td).
  - The obfuscated program $\tilde{CC}\leftarrow \text{Obf}CC\left[f,s,\left(\text{sk},m\right)\right]$, with *f* being the QFHE decryption function; the sender sends pk, *c*_td,_ and $\tilde{CC}$ to the recipient.
(2) The recipient guesses a value of *y* and sends it encoded via the CDS protocol.
(3) The sender responds with a message encrypted via the CDS protocol so that if *y* = *t*, it returns the value *s*. Alternatively returns ⊥.

Intuitively, the above procedure offers commitment since the message in the obfuscated program is uniquely defined and secret since the receiver cannot correctly guess the td value, despite a negligible probability. The simulator can also output (sk, *m*) and simulate the sender's optics. After receiving the first message, it homomorphically calculates the sender's last message using its circuit, entering the encrypted td value and the sender's internal state. The result of the homomorphic calculation is the message encrypted via CDS, whose proposition is correct and returns the value of *s*, encrypted via QFHE. This value is exactly the input required for $\tilde{CC}$ to return the message *m*. In addition, the CC program returns the private sk key together so that the simulator can decrypt the QFHE encrypted messages and generate a valid copy of the *T* communication without using rewinding.

Respectively, the zero knowledge in 4 rounds is proved. We can upgrade the WI protocol to zero knowledge using the above binding technique. In the first round, the verifier sends a zero-value commitment with random *r* (same as the randomness used to generate the keys of the QFHE cryptosystem). Then, we implement the above extraction technique by setting as *m* the randomness *r*. At the end of their interaction, the prover uses the WI protocol to prove that it knows the randomness *r* or *x* ∈ *L* [36, 47].

To defend from malicious attacks where the prover falsifies a valid CDS control witness from the td encryption, we add an SBSH *y* value binding, which can be extracted with low probability, allowing the attack to be reduced QFHE security. At the same time, we check through the CDS protocol that the binding pattern is correctly defined (i.e., the prover includes the randomness of the SBSH binding as part of the witness). For the solution, we use the 3-round postquantum CDS protocol with statistical security on the part of the recipient.

The only issue is that we thought that the verifiers do not interrupt communication and are explainable. For the first problem, we consider two simulators, one for the case that interrupts and one that does not interrupt the interaction. Then, we build a combination simulator that randomly chooses which of the two to use. Watrous' rewinding allows the simulator to rewind until it guesses correctly, without affecting the verifier. On the other hand, to confirm that the verifier is explicable, we add to our protocol a proof of zero knowledge (from the verifier to the prover) that ensures that its messages are honest since the verifier is classical in our protocol, as long as the proof of zero-knowledge is for the NP class [31, 48].

To ensure statistically zero knowledge, however, we must have statistical correctness in the new ZK protocol, and therefore, we need proof of delayed-entry zero knowledge (with statistical correctness). At the same time, the receipt must not exceed three rounds so as not to add extra around the total communication. However, we do not know of any 3-round zero-knowledge protocol (postquantum). However, in our case, a less powerful tool is enough. We can sometimes use simulated (sometimes simulatable) zero knowledge (SSim ZK), where the simulation is possible with a negligible low probability. To use the above tool, we need to configure the security parameters of the other protocols appropriately to compensate for this exponential loss, similar to the SBSH binding pattern. SSim resembles zero knowledge with super polynomial simulation (SPS), with the main difference that in SPS zero knowledge, the simulator runs in hyperpolynomial time, in contrast to SSim zero knowledge, where the simulator runs in polynomial time. Still, there is an exponentially slight chance of success. This difference is of significant importance for our protocol since, basically, we cannot rewind the state of the verifier, and therefore, we require the simulation to be linear.

## 5. Conclusions

This work introduced an innovative blockchain model based on Hyperledger Fabric, which uses Quantum Homomorphic Encryption and Quantum Zero-Knowledge Arguments. The music files are implemented as NFTs, which activate smart contracts. At the same time, through intelligent artificial intelligence algorithms, recommendations are made for the effectiveness in securing the copyrights of the creators of the music industry. As it turned out experimentally, this process can assist in implementing a technologically aware system that can provide a powerful solution to a real modern problem. This is a sophisticated system that is proposed for the first time in the literature.

And while the proposed system presents exceptionally high levels of security, its implementation requires processing all available data before making recommendations, which is not immediately feasible, especially in substantial digital music repositories. Another problem is that they cannot generate recommendations for users who do not have standard ratings with other users and, respectively, cannot suggest items that have not yet been rated by someone (cold start). Finally, they are vulnerable to the problem of overspecialization because they cannot generalize the data they process.

The above weaknesses are also immediate issues for future investigation, which will promote the system's reliability to a much higher degree. The aim is to protect the interests of music producers and the general protection of the music industry's copyright.

## Data Availability

The data used in this study are available from the author upon request.

## References

[1] Orzan M., Orzan G., Spiridon G., Neacsu T. Informatic online system for dissemination of scientific research results for intelectual property rights.

[2] Gonzalez E. J. S., McMullen K. The design of an algorithmic modal music platform for eliciting and detecting emotion.

[3] Zhao Z.-Y., Wang C.-D., Zheng P.-J., Gong Q., Huang K.-W., Lai J.-H. Music sharing platform based on sina app engine.

[4] Amelin R., Arkhipov V., Channov S., Dobrobaba M., Naumov V., Alexandrov D. A., Boukhanovsky A. V., Chugunov A. V., Kabanov Y., Koltsova O., Musabirov I. (2019). Prospects of blockchain-based information systems for the protection of intellectual property. *Communications in Computer and Information Science*.

[5] Kim Y., Kim K.-H., Kim J.-H. Power trading blockchain using hyperledger fabric.

[6] Leal F., Veloso B., Malheiro B., Burguillo J. C., Chis A. E., González-Vélez H. (2021). Stream-based explainable recommendations via blockchain profiling. *Integrated Computer-Aided Engineering*.

[7] Yue Y., Fu X. Research on medical equipment supply chain management method based on blockchain technology.

[8] Bakhytzhan B., Magauiya A., Tuktibayeva M., Gaukhar T. The use of blockchain technology in the field of digital music.

[9] Chohan U. W. (2021). Non-fungible Tokens: blockchains, scarcity, and value. *SSRN Electronic Journal*.

[10] Androulaki E., Barger A., Bortnikov V. Hyperledger fabric.

[11] Nasir Q., Qasse I. A., Abu Talib M., Nassif A. B. (2018). Performance analysis of hyperledger fabric platforms. *Security and Communication Networks*.

[12] Takahashi H., Lakhani U. Voting blockchain for high security NFT.

[13] Yu X., Shu Z., Li Q., Huang J. (Oct. 2021). BC-BLPM: a multi-level security access control model based on blockchain technology. *China Communications*.

[14] Hassan A., Ali M. I., Ahammed R., Khan M. M., Alsufyani N., Alsufyani A. (2021). Secured insurance framework using blockchain and smart contract. *Scientific Programming*.

[15] Yousuf S., Svetinovic D. Blockchain technology in supply chain management: preliminary study.

[16] Li Y., Wei J., Yuan J., Xu Q., He C. (2021). A decentralized music copyright operation management system based on blockchain technology. *Procedia Computer Science*.

[17] Casale-Brunet S., Ribeca P., Doyle P., Mattavelli M. Networks of Ethereum non-fungible Tokens: a graph-based analysis of the ERC-721 ecosystem.

[18] Ito K., O’Dair M., Treiblmaier H., Beck R. (2019). A critical examination of the application of blockchain technology to intellectual property management. *Business Transformation through Blockchain*.

[19] Gürkaynak G., Yılmaz İ., Yeşilaltay B., Bengi B. (Aug. 2018). Intellectual property law and practice in the blockchain realm. *Computer Law & Security Report*.

[20] Hyperledger – Open Source Blockchain Technologies (2022). https://www.hyperledger.org/.

[21] Ampel B., Patton M., Chen H. Performance modeling of hyperledger sawtooth blockchain.

[22] Živić N. Distributed ledger technology for automotive production 4.0.

[23] Chang Y., Fang C., Sun W. (2021). A blockchain-based federated learning method for smart healthcare. *Computational Intelligence and Neuroscience*.

[24] Aleksieva V., Valchanov H., Huliyan A. Implementation of smart-contract, based on hyperledger fabric blockchain.

[25] Alshalali T., M’Bale K., Josyula D. Security and privacy of electronic health records sharing using hyperledger fabric.

[26] Xenya M. C., Quist-Aphetsi K. Decentralized distributed blockchain ledger for financial transaction backup data.

[27] Park W.-S., Hwang D.-Y., Kim K.-H. A TOTP-based two factor Authentication scheme for hyperledger fabric blockchain.

[28] Zhang T. (2000). Association rules. *Knowledge Discovery and Data Mining. Current Issues and New Applications*.

[29] Velasco C., Colomo-Palacios R., Cano R. (2020). Neural distributed ledger. *IEEE Software*.

[30] Behera S., Prathuri J. R. Application of homomorphic encryption in machine learning.

[31] Mohan M., Devi M. K. K., Prakash V. J. Homomorphic encryption-state of the art.

[32] Kim J., Yun A. (2021). Secure fully homomorphic authenticated encryption. *IEEE Access*.

[33] Ahmed H., Glasgow J. (2012). Swarm intelligence : concepts , models and applications technical report 2012-585. https://www.semanticscholar.org/paper/Swarm-Intelligence-%3A-Concepts-%2C-Models-and-Report-Ahmed-Glasgow/116b67cf2ad2c948533e6890a9fccc5543dded89.

[34] Abdelgaber N., Nikolopoulos C. Overview on quantum computing and its applications in artificial intelligence.

[35] Ferrari D., Cacciapuoti A. S., Amoretti M., Caleffi M. (2021). Compiler design for distributed quantum computing. *IEEE Transactions on Quantum Engineering*.

[36] Zhang H., Ji Z., Wang H., Wu W. (2019). Survey on quantum information security. *China Communications*.

[37] Singh J., Singh M. Evolution in quantum computing.

[38] Vuckovic J., Yoshie T., Loncar M., Mabuchi H., Scherer A. Nano-scale optical and quantum optical devices based on photonic crystals.

[39] Zhao X., Si Y.-W. NFTCert: NFT-based certificates with online payment gateway.

[40] Muthe K. B., Sharma K., Sri K. E. N. A blockchain based decentralized computing and NFT infrastructure for game networks.

[41] Erturk E., Dogan M., Kadiroglu U., Karaarslan E. NFT based fundraising system for preserving cultural heritage: heirloom.

[42] Jiang C., Ru C. Application of blockchain technology in supply chain finance.

[43] Tang P., Wang W., Lou J., Xiong L. (2021). Generating adversarial examples with distance constrained adversarial imitation networks. *IEEE Transactions on Dependable and Secure Computing*.

[44] Worthington H., McCrea R. S., King R., Griffiths R. A. (2019). Estimation of population size when capture probability depends on individual states. *Journal of Agricultural, Biological, and Environmental Statistics*.

[45] Ahmed M., Reno S., Akter N., Haque F. Securing medical forensic system using hyperledger based private blockchain.

[46] Hui Y., Zesong L. Research on real-time analysis and hybrid encryption of big data.

[47] Knight P. L. Quantum communication and quantum computing.

[48] Shen T., Wang F., Chen K., Wang K., Li B. (2019). Efficient leveled (multi) identity-based fully homomorphic encryption schemes. *IEEE Access*.

